# Sox2 Is an Androgen Receptor-Repressed Gene That Promotes Castration-Resistant Prostate Cancer

**DOI:** 10.1371/journal.pone.0053701

**Published:** 2013-01-11

**Authors:** Steven Kregel, Kyle J. Kiriluk, Alex M. Rosen, Yi Cai, Edwin E. Reyes, Kristen B. Otto, Westin Tom, Gladell P. Paner, Russell Z. Szmulewitz, Donald J. Vander Griend

**Affiliations:** 1 Committee on Cancer Biology, The University of Chicago, Chicago, Illinois, United States of America; 2 Committee on Immunology, The University of Chicago, Chicago, Illinois, United States of America; 3 Department of Pathology, The University of Chicago, Chicago, Illinois, United States of America; 4 Department of Medicine, Section of Hematology/Oncology, The University of Chicago, Chicago, Illinois, United States of America; 5 Department of Surgery, Section of Urology, The University of Chicago, Chicago, Illinois, United States of America; Northwestern University, United States of America

## Abstract

Despite advances in detection and therapy, castration-resistant prostate cancer continues to be a major clinical problem. The aberrant activity of stem cell pathways, and their regulation by the Androgen Receptor (AR), has the potential to provide insight into novel mechanisms and pathways to prevent and treat advanced, castrate-resistant prostate cancers. To this end, we investigated the role of the embryonic stem cell regulator Sox2 [SRY (sex determining region Y)-box 2] in normal and malignant prostate epithelial cells. In the normal prostate, Sox2 is expressed in a portion of basal epithelial cells. Prostate tumors were either Sox2-positive or Sox2-negative, with the percentage of Sox2-positive tumors increasing with Gleason Score and metastases. In the castration-resistant prostate cancer cell line CWR-R1, endogenous expression of Sox2 was repressed by AR signaling, and AR chromatin-IP shows that AR binds the enhancer element within the Sox2 promoter. Likewise, in normal prostate epithelial cells and human embryonic stem cells, increased AR signaling also decreases Sox2 expression. Resistance to the anti-androgen MDV3100 results in a marked increase in Sox2 expression within three prostate cancer cell lines, and in the castration-sensitive LAPC-4 prostate cancer cell line ectopic expression of Sox2 was sufficient to promote castration-resistant tumor formation. Loss of Sox2 expression in the castration-resistant CWR-R1 prostate cancer cell line inhibited cell growth. Up-regulation of Sox2 was not associated with increased CD133 expression but was associated with increased FGF5 (Fibroblast Growth Factor 5) expression. These data propose a model of elevated Sox2 expression due to loss of AR-mediated repression during castration, and consequent castration-resistance via mechanisms not involving induction of canonical embryonic stem cell pathways.

## Introduction

Relapse of malignant prostate cancer after hormone therapy is a significant clinical problem and new strategies are needed to prevent and treat castration-resistant prostate cancers. Androgen deprivation therapy (ADT) has been the mainstay of prostate cancer treatment since the discovery by Charles Huggins and Clarence Hodges in 1941 that castration significantly aided patients with advanced prostate cancer [1]. However, there is inevitable disease progression due to the growth of castrate-resistant prostate cancer cells. There are a series of mechanisms for the development of castration-resistant prostate cancer (CRPC), most of which center on the Androgen Receptor (AR) [2]. Thus, inhibiting intracellular AR signaling within prostate cancer cells has been a major focus of prostate cancer research, resulting in a variety of chemical inhibitors targeting AR signaling which are used in the clinic [3]. Unfortunately, while all of these inhibitors produce an initial therapeutic response, this is commonly followed by relapse and disease progression.

The recent discoveries of somatic cell reprogramming using defined genes to create induced pluripotent stem cells (iPSCs) profoundly demonstrates that the expression of a few stem cell genes are capable of provoking large scale changes in gene expression and cell behavior, many of which are properties of malignant cells [4]. Indeed, such stem cell reprogramming factors are established oncogenes (c-Myc and Klf4) or are emerging as oncogenes (Sox2, Oct4, and Nanog) in a variety of cancers [5], [6], [7]. The Sox2, Oct4, and Nanog transcription factors comprise the core embryonic stem cell transcription factor machinery and are essential toward maintaining pluripotency and preventing differentiation [8]. In studies using cell lines, these genes not only promote cell proliferation and survival, but also impair normal differentiation processes; both of which are hallmarks of tumorigenesis and disease progression [5], [7], [9], [10], [11], [12], [13], [14], [15]. In some cases, expression of such genes is thought to mark rare cancer stem/initiating cells [12], [16]. Thus, the function of these transcription factors in adult cancer cells is thought to inhibit differentiation and promote stem cell pluripotency and survival mechanisms similar to their essential function in embryonic stem cells.

Sox2 [SRY (sex determining region Y)-box 2] is a transcription factor that is essential for maintaining the survival and pluripotency of undifferentiated embryonic stem cells, and has an emerging role as an epigenetic reprogramming factor and oncogene [5], [17], [18], [19], [20]. In human embryonic stem cells Sox2 regulates the expression of 1259 genes, many of which are co-regulated with Oct4 and/or Nanog [21]. In the prostate, Sox2 expression has been observed in cells within the basal-epithelial cell layer of normal glandular epithelia [22], and in prostate tumors [22], [23]. The expression of Sox2 in prostate tumors has been thought to promote a more aggressive tumor phenotype by promoting a “stem-cell like” tumor phenotype. Indeed, gene array expression analyses showed that an iPS cell-like signature is present within a portion of benign and aggressive prostate tumors, and this signature confers a worse disease prognosis [24]. Our group has previously identified Sox2 as being highly expressed in normal prostate epithelial cells when compared to epithelial cells from the seminal vesicles: an organ with similar function and developmental origin which, unlike the prostate, is rarely a site of tumorigenesis [25]. Collectively, these data lend to the hypothesis that Sox2 functions in adult normal and malignant epithelial cells to regulate the expression of many of the same gene targets regulated by Sox2 within human ES cells, thereby promoting a less differentiated embryonic stem cell tumor phenotype; moreover Sox2 expression could be restricted to rare cancer stem/initiating cells that confer a worse disease prognosis. Alternatively, Sox2 could have a vastly different function in adult epithelial cells. These data also suggest that Sox2 may promote castration-resistance by decreasing the dependence of prostate cancer cells on AR signaling for their growth and survival.

Here we show that Sox2 is expressed in the majority of normal prostate basal epithelial cells, and is uniformly expressed in a subset of prostate tumors. Moreover, Sox2 is expressed in the vast majority of castration-resistant metastatic prostate lesions. In normal prostate epithelial cells, human embryonic stem cells, and castration-resistant prostate cancer cells, ligand activation of AR promotes a decrease in Sox2 expression, which is a result of direct binding of the AR to the Sox2 cis-enhancer region. Expression of Sox2 is also increased within prostate cancer cells that are resistant to the anti-androgen MDV3100. In castration-sensitive prostate cancer cells, Sox2 expression is sufficient to promote castration-resistant tumor formation. The expression of Sox2 in prostate cancer cells, however, does not result in changes in differentiation-specific PSA expression, an increase in CD133-positive putative cancer stem/initiating cells, or the expression of known human Embryonic Stem Cells (hESC)-associated Sox2 target genes. Thus, Sox2 appears to promote castration resistance via mechanisms that do not involve the re-expression of embryonic stem cell Sox2-target genes or increases in rare tumor stem/initiating cell populations.

## Materials and Methods

### Cell Lines and Materials

R1881 and Actinomycin D were purchased from Sigma-Aldrich (St. Louis, MO), and MDV3100 was purchased from Selleck Chemicals (Houston, TX), and stored at −20 in Ethanol. All human prostate cancer cell lines were grown as previously described [26], [27]. All cultures were routinely screened for the absence of mycoplasma contamination using the ATCC Universal Mycoplasma Detection Kit (Manassas, VA). PC-3, VCAP, and NCCIT cell lines were purchased from ATCC, and the Du145, LNCaP, LAPC-4, C4-2, CWR22Rv1, CWR-R1, MDA-PCa2B, and E006AA cell lines were generously provided by Dr. John Isaacs at The Johns Hopkins University and have been previously characterized [28]. The human embryonic stem cell line WA01(H1) was acquired from WiCell (Madison, WI) and cultured using the feeder-independent protocol using mTeSR1 media (Stem Cell Technologies; Vancouver, B.C.). ES cells were used within ten passages of thawing. Secreted total PSA and Testosterone were measured using Roche Elecsys Total Prostate-Specific Antigen (PSA) and Testosterone (T) Assays. Cell growth was measured using the Vybrant MTT Cell Proliferation Assay Kit (Invitrogen/Molecular Probes; Eugene, OR).

Lentiviral Sox2, AR, and Control vectors (pReceiver-Lv105) were purchased from GeneCopoeia (Rockville, MD). Lentiviral tetracycline transactivator (LV-rtTA) and inducible lentiviral Sox2 vector from the Hochedlinger lab were purchased via Addgene [29]. When necessary, Sox2 expression was induced using 1 ug/mL Doxycycline (Sigma). Knockdown of Sox2 expression using shRNA expression was achieved using the anti-human Sox2 shRNA gene set, which consists of 4 different lentiviral targeting sequences against Sox2 and a non-silencing control [HSH017628-HIVH1(Sox2) and CSHCTR001-HIVH1 (control) vectors containing a GFP and puromycin-resistance components; Genecopoeia]. High-titer lentivirus was made by co-transfecting with ViraPower Lentivrial packaging mix (Invitrogen; Grand Island, NY) and Lenti-X Concentrator (Clontech; Mountain View, CA) according to manufacturer's instructions.

Non-malignant epithelial cultures were established from fresh human prostate tissues acquired from surgical specimens as previously described [25]. These tissues were acquired under an expedited protocol approved by the University of Chicago Institutional Review Board (IRB). Tissue samples were managed by the University of Chicago Human Tissue Resource Center core facility; the need for patient consent was waived as acquired samples were de-identified. 4 mm biopsy punches of non-tumor tissue were taken from prostate and seminal vesicle tissues of patients undergoing radical prostatectomy; half of this tissue was fixed and analyzed by a pathologist to confirm the absence of tumor. Dissociation of prostate tissue and growth of epithelial cells has been previously described [30], [31], and the same methods were used to establish matched prostate (PrEC) and seminal vesicle (SVEC) epithelial cell cultures. Cultures were grown using Keratinoctye Serum-Free Defined media supplemented with growth factors (GFs) (standard K-SFM, Invitrogen Life Technologies) and can be cultured up to 8 passages before notable cellular senescence [32]. For our experiments, all cultures were analyzed at or before their fourth passage.

### Western Blotting, Immunohistochemistry, and Immunofluorescence

Whole-cell lysates collected from 100,000 cells were used per lane. Antibodies used were: anti-AR (N-20, Santa Cruz; Santa Cruz, CA); anti-Beta Actin (Sigma-Aldrich); anti-ΔNp63 (4A4, Santa Cruz); anti-Sox2 (D6D9 XP, Cell Signaling Technology; Beverly, MA); anti-Nanog (D73G4 XP, Cell Signaling Technology); anti-Oct4 (C30A3, Cell Signaling Technology); and anti-glyceraldehyde-3-phosphate dehydrogenase (GAPDH, Cell Signaling Technology). Secondary horseradish peroxidase-conjugated antibodies were from Cell Signaling Technologies, and HRP detected using SuperSignal West Femto Chemiluminescent Substrate (Peirce/Thermo Scientific; Rockford, IL). Alternatively, secondary antibodies from Rockland (Gilbertsville, PA) were used and data captured using a Licor Odyssey system (Lincoln, NE).

Immunostaining for Sox2 (D6D9 XP, Cell Signaling Technology; rabbit monoclonal) was performed on formalin-fixed, paraffin-embedded (FFPE) sections managed either by the University of Chicago Human Tissue Resource Core facility or the Northwestern University/University of Chicago Prostate SPORE program and their Specimen Procurement Program. After deparaffinization and rehydration, tissues were treated with antigen retrieval buffer (S1699 from DAKO; Glostrup, Denmark) in a steamer for 20 minutes. Anti-Sox2 antibody (1∶25 dilution) was applied for 1 hour at room temperature in a humidity chamber. Following TBS wash, the antigen-antibody binding was detected with Envision+system (DAKO, K4001 for mouse primary antibodies) and DAB+Chromogen (DAKO, K3468). Tissue sections were briefly immersed in hematoxylin for counterstaining and were cover-slipped. Tissues were analyzed by a trained Genitourinary Pathologist and scored on percentage of cells with positive nuclear staining (0 = no staining; 1 = 1–10% positive cells; 2 = 11–50% positive cells; and 3 = >50% positive cells); as well as the intensity of staining (0 = no staining; 1 = weak staining; 2 = moderate staining; 3 = strong staining). For images, slides were digitized using a Pannoramic Scan Whole Slide Scanner (Cambridge Research and Instrumentation; Hopkinton, MA) and images captured using the Pannoramic Viewer software version 1.14.50 (3DHistech; Budapest, Hungary).

For immunofluorescence, tissues were deparaffinized, rehydrated, and treated with antigen retrieval buffer. Antibody binding of Sox2 (D6D9, Cell Signaling Technology; Alexa-Fluor 555-conjugated, 1∶50 dilution in TBST) and p63 (4A4, Santa Cruz Biotechnology; Alexa-Fluor 647 conjugated, 1∶50 dilution in TBST) were conducted for 1 hr at room temperature. Tissues were counter-stained with DAPI and mounted using Fluoromount-G (Southern Biotech, Birmingham, AL). Fluorescent images were captured using a Leica TCS SP2 AOBS Laser Scanning Confocal microscope.

### Quantitative Real Time PCR (Q-RT-PCR) and PCR Array Analyses

RNA was purified using the Qiagen RNeasy Mini Kit with the optional DNAse digestion kit (Qiagen, Valencia, CA) and quality tested using an Agilent Bioanalyzer 2100 (Agilent Technologies, Santa Clara, CA). For standard Q-RT-PCR, extracted RNA was converted to cDNA by reverse transcription using SuperScript® III Reverse Transcriptase (Invitrogen). Levels of Sox2, PSA, FGF5 and GAPDH transcript were quantified using *Power* SYBR® Green Master Mix (Invitrogen) using custom primers for Sox2 [5′-AACCCCAAGATGCACAACTC-3′ (forward), 5′-CGGGGCCGGTATTTATAATC-3 (reverse)]; PSA [5′-TCATCCTGTCTCGGATTGTG-3′ (forward), 5′-ATATCGTAGAGCGGGTGTGG-3′ (reverse)]; FGF5 [5′-AGTCAATGGATCCCACGAAGC-3′ (forward), 5′-TGAACTTGGCAG TTGCATGGA-3′ (reverse)]; and GAPDH [5′-GAGTCAACGGATTTGGTCGT-3′ (forward), 5′-TTGATTTTGGAGGGATCTCG-3′ (reverse)]. Standard curves were used to assess primer efficiency and average change in threshold cycle (ΔCT) values was determined for each of the samples relative to endogenous GAPDH levels and compared to vehicle control (ΔΔCT). Experiments were performed in triplicate to determine mean standard error, and student's t-tests performed with normalization to control to obtain p-values.

For the Human Embryonic Stem Cell PCR Arrays, the RT^2^ First Strand Kit (SA Biosciences; Valencia, CA) was used to reverse transcribe the RNA to cDNA. RT^2^ qPCR Master Mix (SA Biosciences) and Human Embryonic Stem Cell RT^2^ Profiler™ PCR Arrays (Cat# PAHS-081, SA Biosciences) were used to examine the transcripts of 84 key genes involved in the maintenance of pluripotency and the self-renewal status of embryonic stem cells. Arrays were performed with biological replicates in triplicate for each condition. The RT^2^ Profiler™ PCR Array Data Analysis software was used to assess average change in threshold cycle (ΔCT) values for each of the samples relative to vehicle control (ΔΔCT), determine standard error and obtain p-values via student's t-test.

### AR Chromatin Immunoprecipication

Chromatin-IP protocols were adapted from previously reported methods [33]. Cells were grown to 70–90% confluence and treated for 24 hours with 1 nM R1881. Cells were fixed with 1% formaldehyde at room temperature for 15 minutes; the crosslinking was quenched with 0.125M Glycine in PBS for 15 minutes at room temperature and washed with ice-cold PBS. After cells were scraped off in PBS plus 1× protease inhibitor cocktail (Roche Molecular Biochemicals; Penzberg, Germany), cell pellets were collected by centrifugation and cell nuclei extracted by centrifugation at 14,000 RPM at 4°C for 15 minutes. Cell nuclei were re-suspended and washed in micrococcal nuclease buffer (Tris [pH 7.4] 10 mM, NaCl 15 mM, KCl 60 mM, Spermine 0.15 mM, Spermidine 0.5 mM, CaCl2 1 mM) then incubated with 10,000 U of micrococcal nuclease for 20 minutes at 37°C with gentle thermomixing. Nuclei were then collected and re-suspended in RIPA-PIC buffer [150 mM sodium chloride, 1.0% Igepal CA-630 (Sigma-Aldrich), 0.5% sodium deoxycholate, 0.1% SDS, 50 mM Tris, pH 8.0/1× protease inhibitor cocktail (Roche)] and sonicated (Fisher Scientific; Hampton, NH; model FB-120 Sonic Dismembrator). Sonicated chromatin extracts were then pre-cleared overnight at 4°C by incubation with protein G-agarose beads treated with Normal Rabbit IgG (Cell Signaling Technology), and chromatin fragments were immunoprecipitated with specific antibodies overnight at 4°C. For a 1-ml diluted chromatin solution, 50 µg of the following antibodies were used: Normal Rabbit IgG (Cell Signaling Technology) as a negative control; Histone H3 (Abcam; Cambridge, MA) as a positive control; and AR (N-20, Santa Cruz,) for the experimental conditions. Beads were then collected and washed in TSE I buffer [0.1% TritonX-100, 2 nM EDTA, 150 mM NaCl, 20 mM Tris-HCl (pH 8.1)], TSE II buffer [0.1% SDS, 1% TritonX-100, 2 mM EDTA, 500 mM NaCl, 20 mM Tris-HCl(pH 8.1)], Brown Buffer III [0.25 LiCl, 1.0% Igepal CA-630 (Sigma-Aldrich; St. Louis, Mo), 1% deoxycholate, 1 mM EDTA, 10 mM Tris-HCL(pH 8.1)], and twice with TE Buffer [1 mM EDTA, 10 mM Tris-HCL(pH 8.1)]. The immunoprecipitated chromatin fragments were eluted off of the agarose beads using Brown Elution Buffer [1% SDS, 0.1M NaHCO3, 1 mM DTT, 1× protease inhibitor cocktail (Roche)] and incubated for 10 minutes at 95°C with vigorous thermomixing. Eluted chromatin and inputs was then incubated overnight at 65°C with gentle thermomixing to reverse crosslinks. Samples were then treated with 2 µg RNaseA (Novagen; Darmstadt, Germany) at 37°C for 15 minutes, then with 2 µg Proteinase K at 55°C for 30 minutes (Cell Signaling Technology). Finally, immunoprecipiated DNA fragments were extracted by Phenol-Chloroform (Sigma-Aldrich) extraction and subjected to Q-RT-PCR using commercially available SimpleChIP™ Human Sox2 cis-enhancer promoter primers (Cell Signaling Technology).

### In Vivo Tumor Formation

All animal studies were carried out in strict accordance with the recommendations in the Guide for the Care and Use of Laboratory Animals of the National Institutes of Health. The protocol was approved by the University of Chicago Institutional Animal Care and Use Committee (IACUC, protocol numbers 72066 and 72231). All surgery was performed under Ketamine/Xylazine anesthesia, and all efforts were made to minimize suffering. *In vivo* tumor formation of LAPC-4 and CWR-R1 cells were conducted via a sub-cutaneous inoculation of one million cells in 4–6 week old male athymic nude mice (Harlan; Indianapolis, IN) using a 75% Matrigel and 25% HBSS solution (BD Biosciences). To measure tumor take in a castrated host, host mice were surgically castrated one week prior to cell inoculation. To measure progression to castration resistance, animal hosts were castrated when tumors reached 0.5 cm^3^. To analyze circulating total PSA from the tumor implant and to confirm testosterone depletion in castrated hosts, blood was drawn via cardiac puncture at the experimental endpoint.

### Flow Cytometry

All antibody incubations, washes, and flow cytometric analyses were performed using ice cold cell sorting buffer (1× PBS, 0.5% bovine serum albumin (BSA), 2 mmol/L EDTA) in minimal light using previously reported protocols [26], [31]. Cells were stained with Live/Dead Fixable Green Dead Cell Stain Kit (Invitrogen) per the manufacturer's instructions. After washing, cells were incubated with FcR Blocking Reagent (Miltenyi Biotec; Cologne, Germany, 1∶20 dilution for 10 minutes), and then labeled with CD133/2 (293C3)-APC–conjugated human monoclonal antibody (Miltenyi Biotec) or isotype control mouse IgG2b-APC (Miltenyi Biotec) at a 1∶10 dilution for 30-min. The cells were then washed and fixed for 15 minutes with 3.2% Ultra Pure EM Grade Formaldehyde (Polysciences, Inc.; Warrington, PA). After fixation, the cells were washed and stained with FXCycle Violet (Invitrogen, 1∶1000 dilution). Analysis was conducted on a Becton Dickinson LSR II, and a minimum of 250,000 counts was acquired for each experimental condition. FACSDiVa Software was used for data acquisition, and FlowJo software was used for analysis.

### Floating Spheroid Culture Assay

*In vitro* floating spheroids were grown from 1×10^5^ LAPC-4 and LNCaP cells transduced with either lentiviral Sox and GFP ConFtrol vectors [pReceiver-Lv105; GeneCopoeia (Rockville, MD)]. Cells were cultured on Ultra-Low attachment 25 cm^2^ cell culture flasks (Corning; Corning, NY) and grown in suspension for 5 days. Spheroids formed after 5 days were transferred to 10 cm^2^ dishes (Corning; Corning, NY) for 24 hours to allow attachment. Spheroids were then stained with crystal violet solution (Sigma-Aldrich), rinsed, and counted. Sphere formation was performed in triplicate.

### Statistical Analyses

In all instances data was reproduced using multiple biological and technical replicates. Data was analyzed using SigmaPlot 11.0 software using a one way ANOVA with Pair-wise Multiple Comparison Procedures (Holm-Sidak method).

## Results

### Sox2 is Expressed in Normal Prostate Basal Epithelial Cells and in a Subset of Prostate Tumors

**Figure 1:**
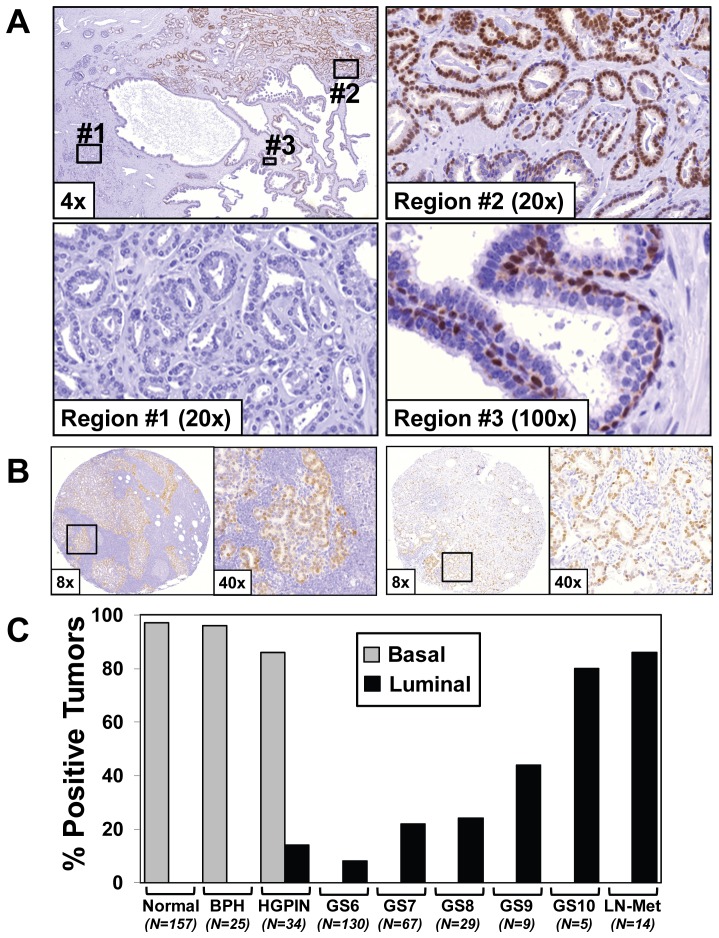
Sox2 is uniformly expressed in a subset of hormone naïve prostate tumors and castration-resistant metastases. **A**) Immunohistochemical staining of Sox2 demonstrating representative nuclear basal epithelial staining (dark red) in normal glands (Region #3), and two distinct tumor regions that are either uniformly Sox2-positve (Region #2) or Sox2-negative (Region #1). **B**) Expression of Sox2 in representative castration-resistant metastatic lesions. The box in the 8× magnification corresponds to the region shown in the 40× image. **C**) Percentage and distribution of Sox2 expression among prostate disease states. In normal, BPH, and HGPIN tissues, Sox2 is expressed in basal-epithelial cells (Grey Bars). Positive expression is defined by more than 50% of the cancer cells positive with a relative intensity of 1 or more on a scale of 0–3. In cancer tissues and metastases, Sox2 is either uniformly expressed or absent, and the percentage of Sox2-positive tumors increases with Gleason Score (Black Bars). BPH: Benign Prostatic Hyperplasia; HGPIN: high-grade prostatic intraepithelial neoplasia; GS: Gleason Score (N = number of individual patient specimens analyzed).

To evaluate the prevalence and pattern of Sox2 protein expression in the prostate, we conducted an immunohistochemical evaluation of a series of human prostate tissues and tumors. These analyses show that in normal and benign hyperplastic (BPH) prostate tissues Sox2 expression is restricted to basal epithelial cells (**Figure 1A**). Analyses of prostate tumors demonstrates that Sox2 is either uniformly expressed or uniformly absent (**Figure 1A**). The majority (12 of 14) of castration-resistant metastases analyzed also express Sox2 (**Figure 1B and C**). The percentage of Sox2-positive tumors increases with Gleason Score, with a small subset of favorable-prognosis Gleason Score 6 tumors and the majority of poor-prognosis Gleason Score 10 and castration-resistant metastases positive (**Figure 1C**). Interestingly, analyses of pre-malignant prostatic intra-epithelial neoplasia (PIN) lesions documented a mixed basal and luminal epithelial cell staining (**Figure 1C**). These tumor data are consistent with the observation made by Jia et al. using a different Sox2 antibody showing that expression correlates with Gleason Grade [**23**]. Our data contrasts with this report, however, as we observe strong basal-epithelial staining in non-malignant tissues, as well as uniform strong Sox2 expression in a small percentage of tumors, rather than a graded increase in staining intensity [23]. These data document that Sox2 is uniformly expressed in a subset of prostate tumors, and indicates that Sox2 expression confers a unique tumor sub-type which does not appear to be restricted to rare cancer initiating/stem-like cells in prostate tumors.

### Sox2 is Co-Expressed Within a Portion of p63-Positive Basal Epithelial Cells

**Figure 2:**
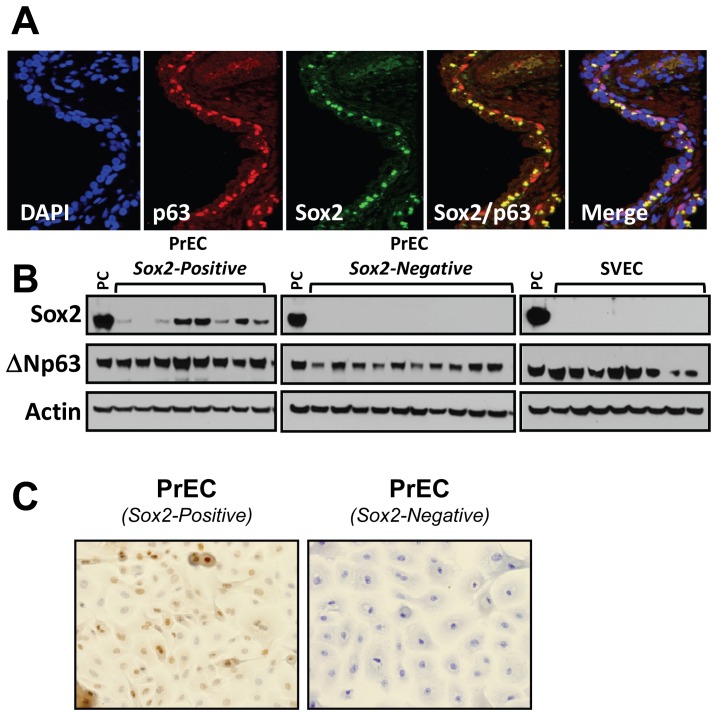
Sox2 is expressed in the majority of normal basal-epithelial cells, and cultures derived from prostate epithelial cells (PrECs) are either uniformly Sox2-Positive or Negative. **A**) Immunofluorescent co-staining of Sox2 (green) with the basal-specific marker p63 (red), showing that 75% of normal basal-epithelial cells are positive for both Sox2 and p63 (yellow) while the remaining 25% are Sox2-negative (red). DAPI staining highlights nuclei (representative images of normal prostate epithelium acquired from three different individual patient specimens). **B**) Western blotting of a series of patient-derived Prostate (PrEC) and Seminal Vesicle (SVEC) epithelial sell (PrEC) cultures demonstrates that a portion of these cultures express detectable Sox2, while other PrEC and all SVEC cultures do not. **C**) Immunocytochemical staining of Sox2 showing uniform nuclear Sox2 expression in Sox2-positive PrECs and lack of Sox2-positive cells within Sox2-negative PrECs (representative images of three independent experiments).

The expression of Sox2 in AR-negative basal-epithelial cells implies that Sox2 may function to promote the survival of prostate stem and basal transient-amplifying cells, or to maintain them in an undifferentiated state. In the normal prostate, the paracrine interaction between stromal and epithelial cells is androgen dependent, where AR-positive stromal cells produce a series of growth factors, collectively termed “andromedins” which signal to nearby epithelial cells to promote the proliferation of AR-negative, p63-positive basal epithelial cells and the survival of AR-positive/Prostate Specific Antigen (PSA)-positive luminal epithelial cells [34]. To determine what proportion of basal-epithelial cells were Sox2-positive, we conducted co-immunofluorescent staining of Sox2 and p63. These data show that 75% of prostate basal epithelial cells located in the prostatic peripheral zone express both Sox2 and p63, while the remaining 25% of cells express p63 but not Sox2 (**Figure 2A**). This observation implies that Sox2 expression delineates two potential populations of basal epithelial cells; these cells may also have unique phenotypic characteristics and may derive distinct tumors [35].

Based on this, we established a series of non-malignant prostate epithelial cell (PrEC) cultures from freshly dissociated prostate tissue and analyzed them for Sox2 expression. Such PrEC cultures do not express detectable level of AR protein, as they consist of mostly p63-positive transient-amplifying cells, along with minor populations of CD133-positive stem cells, PSCA-positive intermediate cells, and Chromogranin A-positive neuroendocrine cells [26], [31]. As an additional control, we isolated patient-matched Seminal Vesicle epithelial cell (SVEC) cultures using the same methods. Western blotting analyses documented that PrEC cultures were either Sox2-positive or Sox2-negative, and matching SVEC cultures were consistently negative (**Figure 2B**) [25]. The heterogeneous cell populations within such PrEC cultures suggested that Sox2 may be highly expressed in a small subset of cells. Immunocytochemical analyses of Sox2 expression, however, documents that the majority of cells within Sox2-positive PrEC cultures express Sox2; while there are few, if any, detectable Sox2-expressing cells in Sox2-negative PrEC cultures (**Figure 2C**). Thus, in non-malignant PrEC cultures, Sox2 expression is not restricted to a unique cell population such as CD133-positive prostate stem cells.

### Increased Androgen Receptor (AR) Signaling Decreases Sox2 Expression in Normal Prostate Epithelial Cells (PrECs) and Human Embryonic Stem Cells (hESCs)

**Figure 3:**
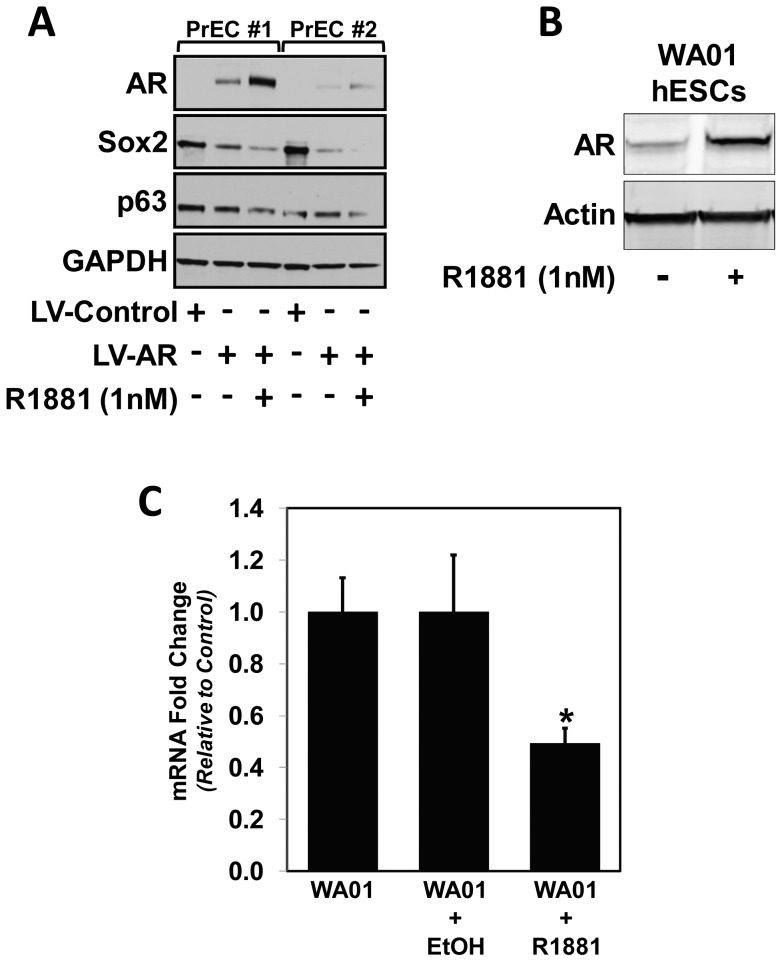
Sox2 is negatively regulated by Androgen Receptor (AR) signaling in prostate epithelial cells (PrECs) and human embryonic stem cells (hESCs). **A**) To test whether AR signaling impacted Sox2 expression, we ectopically expressed AR (LV-AR) and control lentivirus (LV-Control) in two different Sox2-positive PrEC lines (#1 and #2). Western blotting shows that expression and further ligand activation of AR using 1 nM R1881 results in decreased Sox2 protein expression and decreased p63 expression. GAPDH was used as a loading control. **B**) To test whether AR-mediated inhibition of Sox2 expression was specific to PrECs or could also occur in Sox2-positive human ES cells, we treated the WA01(H1) human ES cell line with androgen (1 nM R1881). Western blotting documents detectable endogenous AR expression in hESCs, which increases in response to androgen. β-Actin was used as a loading control. **C**) AR activation in hESCs results in a significant decrease in Sox2 mRNA expression as measured by qPCR (* indicates p<0.05). This was compared to untreated and vehicle treated cells (EtOH).

It has been previously demonstrated experimentally that activation of AR signaling by androgen binding in prostate epithelial cells induces their growth arrest and eventual terminal differentiation into secretory-luminal cells [36], [37], [38]. The expression of Sox2 in basal epithelial cells and the lack of Sox2 expression in non-malignant AR-positive luminal epithelial cells suggested that AR may repress Sox2 expression. Using Sox2-positive PrEC cultures, the response of Sox2 expression to exogenous expression and ligand induction of wild-type AR was evaluated. To accomplish this, PrEC cultures were transduced using a lentiviral construct containing AR cDNA (LV-AR) or an empty control vector (LV-Control) (**Figure 3A**). Indeed, AR expression and further stimulation with physiological levels (i.e. 1.0 nM) of the synthetic androgen R1881 results in decreased expression of both Sox2 and p63 (**Figure 3A**). In the absence of AR protein expression, treatment with R1881 had no effect on Sox2 expression (data not shown); which is consistent with previously published data [28].

To test whether such AR-mediated suppression of Sox2 is unique to prostate cells, we examined the ability of AR signaling to decrease Sox2 expression within the human embryonic stem cell line WA01 (H1). Sox2 is indispensible for maintaining hESC pluripotency and for the generation of induced pluripotent stem cells (iPSCs) [4], [21]. Interestingly, hESCs express detectable levels of endogenous AR, and the expression of AR protein increases with addition of 1 nM R1881 to the media (**Figure 3B**). Such AR stimulation also results in a marked decrease in Sox2 expression (p<0.05) (**Figure 3C**). These data demonstrate that, in pluripotent embryonic stem cells and undifferentiated prostate transit-amplifying cells, Sox2 expression decreases in response to increased AR signaling. This supports a role for Sox2 in maintaining basal epithelial cells in a less-differentiated state, and further supports a role for the AR in promoting the terminal differentiation of non-malignant prostate epithelial cells.

### Increased Androgen Receptor Signaling Decreases Sox2 mRNA and Protein Expression in Castration-Resistant Prostate Cancer (CRPC) Cells

**Figure 4:**
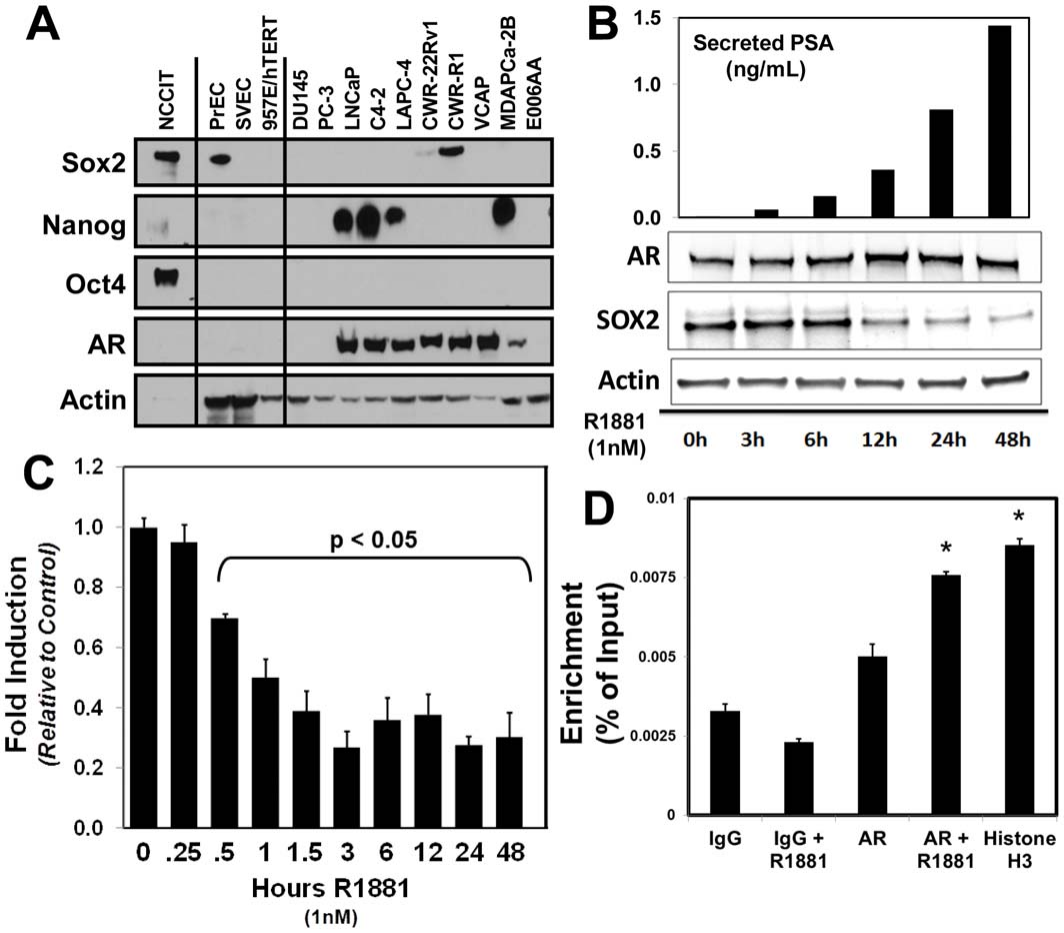
Androgen Receptor (AR) directly represses Sox2 expression in castration-resistant CWR-R1 cells. **A**) Western blot of a panel of non-malignant and prostate cancer cell lines for Sox2, Nanog, Oct4, and AR. β-Actin was used as a loading control. Expression of Sox2 in castration-resistant CWR cells is not accompanied by co-expression of Nanog or Oct4. LNCaP, C4-2B, LAPC-4, and MDA-PCa2B cells expressed detectable Nanog, which is presumably the NanogP8 retrogene (Jeter et al., 2011). The human embryonal carcinoma cell line NCCIT was used as a positive control for Sox2, Nanog, and Oct4 at a 1∶10 dilution. **B**) Decreased expression of Sox2 upon AR stimulation with physiologic levels of androgen (1 nM R1881) in castration-resistant CWR-R1 prostate cancer cells. Protein lysates from cells treated at defined intervals (3–48 hours) after androgen treatment were subjected to western blotting, and accumulation of secreted PSA expressed in the media validates increased AR signaling. **C**) Rapid decrease of Sox2 mRNA in CWR-R1 cells upon AR stimulation as measured by qPCR. Levels at 0.5 hrs and beyond represent a statistically significant decrease in Sox2 mRNA (p<0.05). **D**) AR Chromatin Immunoprecipitation (ChIP) documents direct binding of ligand-activated AR to the Sox2 enhancer region in response to AR stimulation by R1881. CWR-R1 cells were treated with vehicle control or 1 nM R1881, and enrichment of the Sox2 promoter after AR-ChIP was normalized as a percentage of total chromatin input. IgG and Histone H3 served as negative and positive controls, respectively. When compared to total input, both the positive control Histone H3 and ligand-activated AR significantly enriched for the Sox2 enhancer (p<0.05). Data represents three independent experiments.

The observation that AR signaling suppresses Sox2 expression, the increased expression of Sox2 in higher grade prostate tumors and metastases, and the previously documented role of Sox2 in maintaining stem cell survival and pluripotency inferred that Sox2 may serve an important function in promoting prostate cancer progression and castration resistance. Western blot analyses of a panel of normal and malignant prostate cell lines demonstrates that, along with PrEC cultures, the CWR-R1 and CWR-22Rv1 cell lines express detectable Sox2 (**Figure 4A** **and** **Table 1**). These two cell lines were derived from the same parent tumor, and both are castration resistant in that they reliably and robustly form tumors when inoculated into castrated male nude murine hosts [39], [40]. In hES cells, Sox2, Nanog, and Oct4 constitute the core transcriptional machinery controlling stem cell pluripotency, functioning together to regulate the expression of numerous genes involved in maintaining stem cell pluripotency [41], [42]. Both the PrEC and CWR cell lines, however, do not express detectable levels of the Sox2 co-regulators Nanog and Oct4 (**Figure 4A**), indicating that Sox2 may have a unique function and non-stem cell gene targets in normal and malignant adult prostate epithelial cells. Other lines, such as LNCaP and LAPC-4 cells, express detectable levels of Nanog, which has been previously shown to be the NanogP8 retrogene [43].

**Table 1:** Levels of Sox2 mRNA.

Cell Line	Fold Expression^A^
PrEC	1.00±0.12
SVEC	ND
Du145	ND
PC-3	ND
LNCaP	ND
LAPC-4	ND
CWR-22rv1	0.03±0.04
CWR-R1	1.69±0.10
VCaP	ND
NCCIT	5.83±0.01
WA01	23712.06±0.07

A : Values are fold difference compared to PrEC.

ND: Not Detectable (<0.01-fold).

Previous reports in hESCs have demonstrated that the levels of Sox2 expression are an important factor in the ability of Sox2 to maintain pluripotency [19]. Moreover, contrary to our observations, Jia et al. observed detectable Sox2 expression in PC-3, Du145, and LNCaP prostate cancer cells [23]. To resolve this, we quantified the level of Sox2 mRNA expression compared to the embryonal carcinoma cell line NCCIT and hESCs; since Sox2 is an intron-less gene, we added an additional DNAse-digestion step to ensure that no contaminating genomic DNA was present which would confound PCR detection of mRNA. These data show that PrEC and CWR lines express detectable levels of Sox2 (R-1: 1.68-fold higher than PrEC; 22Rv1: 0.026-fold that of PrEC) which are substantially below that of pluripotent NCCIT cells and hESCs (**Table 1**), and the PC-3, Du145, LNCaP, LAPC-4, and VCaP prostate cancer cell lines do not express detectable quantities of Sox2 mRNA.

Similar to PrEC and hESCs, increased AR signaling by the addition of physiologic levels of androgen (i.e. 1 nm R1881) results in a significant decrease in endogenous Sox2 protein (**Figure 4B**); this decrease in Sox2 contrasts with an increase in secreted total secreted PSA detected in the media (**Figure 4B**), which is expected as PSA is an AR-target gene [44]. Nanog levels did not become detectable as Sox2 expression decreased (data not shown). Time course analyses of Sox2 mRNA expression documents that within 30 minutes there is a statistically-significant (p<0.05) decrease in Sox2 mRNA expression within CWR-R1 prostate cancer cells (**Figure 4C**). This rapid decrease in Sox2 mRNA in response to increased AR signaling indicated that AR may be acting directly on the Sox2 promoter. To confirm this, AR-Chromatin Immuno-Precipitation (ChIP) analyses demonstrate that, upon treatment with R1881, AR directly binds to the Sox2 enhancer element (**Figure 4D**).

These findings are novel, since AR ChIP-Seq and ChIP-Chip studies performed by Yu et al., Wang et al., and Urbanucci et al. did not identify Sox2 as a target gene of AR in LNCaP and VCaP cell lines, with the nearest AR binding sites being over 150 kb away from the gene [33], [45], [46]. However, data by Yu et al. and Kim et al. 2010 suggest that the Sox2 promoter is most likely inaccessible to AR due to the locus being silenced and heterochromatinized, as indicated by the presence of the Histone H3 post-translational modification tri-methylated Lysine 27 (H3K27me3) in both LNCaP and VCaP cells, and CpG island methylation in the Sox2 coding sequence and promoter in LNCaP cells [45], [47]. ChiP-Seq analysis of AR in other another cell line with a presumably different epigenetic state, PC3 cells transduced with AR (PC3-AR), performed by Lin et al. identified Sox2 as being an AR target gene [48]. AR was found to bind 5137 bases upstream from the transcriptional start site near the Sox2 promoter enhancer sequence, as well as in the coding sequence (539 from the transcriptional start site) of Sox2, without leading to a detectable increase in mRNA expression in these cells [48]. Together, these data further support the role of AR as a direct transcriptional repressor of Sox2.

### Direct Androgen Receptor-mediated Repression of Sox2 mRNA and Protein Expression can be Reversed by Anti-Androgen Treatment

**Figure 5:**
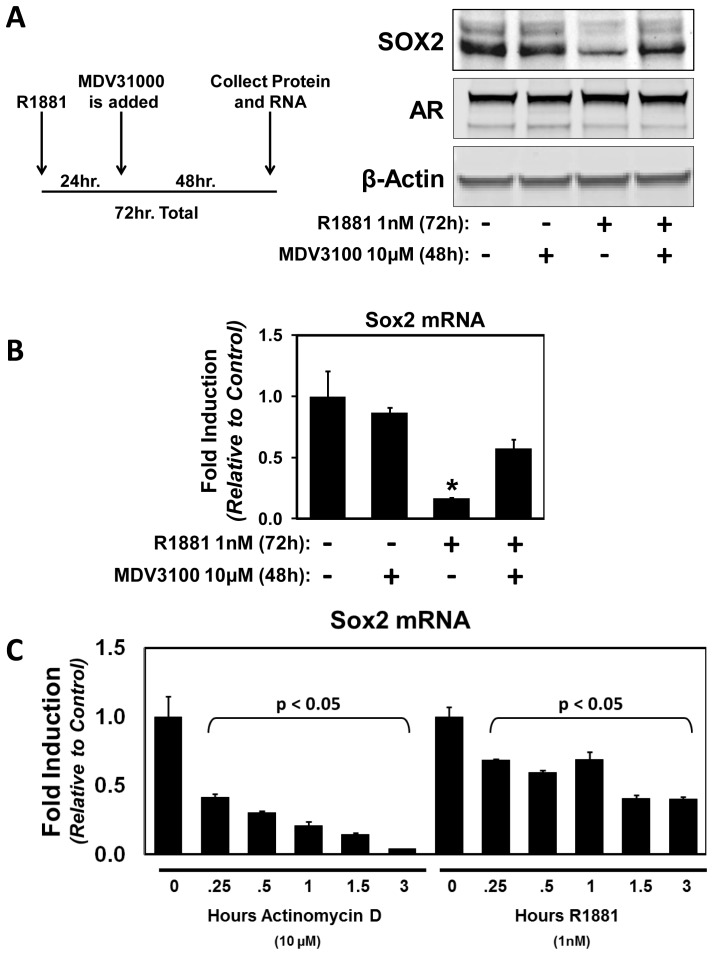
Androgen Receptor-mediated repression of Sox2 expression can be reversed by treatment with the Anti-Androgen MDV3100. **A**) To verify that the decrease of Sox2 protein is specific to AR activation and can be reversed by an AR antagonist, CWR-R1 cells were grown in 1 nm R1881 or vehicle for 24 hrs, and then either 10 µM MDV3100 or vehicle was added to the culture medium for an additional 48 hrs. Western blots show a decrease in Sox2 protein with R1881 that can be returned to basal levels with addition of MDV3100, without any change in AR protein. β-Actin was used as a loading control. A schematic outlines the time frame of these experiments. **B**) A decrease of Sox2 mRNA with R1881 treatment was measured using qPCR (*p<0.05), which was brought back to control levels upon treatment with MDV3100. **C**) A time course of treatment with a known inhibitor of transcription, 10 µM Actinomycin D, yielded a similar rapid decrease of Sox2 mRNA in CWR-R1 cells upon AR stimulation with R1881 as measured by qPCR. Levels at 0.25 hrs and beyond represent a statistically significant decrease in Sox2 mRNA (p<0.05), showing similar kinetics of transcriptional repression with both R1881 and Actinomycin D drug treatments.

Anti-Androgens are AR antagonists that are often used clinically in conjunction with ADT or as a monotherapy to block AR activity in prostate cancer cells [49]. MDV3100 is a FDA-approved second-generation anti-androgen that has been shown to decrease AR nuclear translocation and prevent its DNA binding [50]. Given our observation that AR activation can lead to direct DNA-binding and repression on the Sox2 promoter, we investigated the ability of MDV3100 to override AR-mediated Sox2 transcriptional repression. To verify that the decrease in Sox2 expression is specific to AR activation and can be reversed by antagonization, CWR-R1 cells were grown in 1 nm R1881 or vehicle for 24 hours, and then either 10 µM MDV3100 or vehicle was added to the culture medium for 48 hours. 10 µM MDV3100 has been shown to fully bind and saturate AR both *in vivo* and *in vitro* [51]. MDV3100 treatment reverses androgen-mediated decreases of Sox2 protein and mRNA in CWR-R1 cells 48 hours after a 24 hour pretreatment with R1881 (**Figure 5A** **and** **Figure 5B**); illustrating that these effects are specific to AR activity and that AR-mediated Sox2 repression is reversible. Furthermore, the kinetics of AR activation leading to a decrease in Sox2 mRNA is similar to treatment with the known inhibitor of transcription, Actinomycin D (**Figure 5C**). These data provide further evidence for direct AR mediated-transcriptional repression of Sox2 expression.

### Increased Sox2 Expression is Associated with Acquisition of Resistance to Anti-Androgen in Vitro and Castration in Vivo

**Figure 6:**
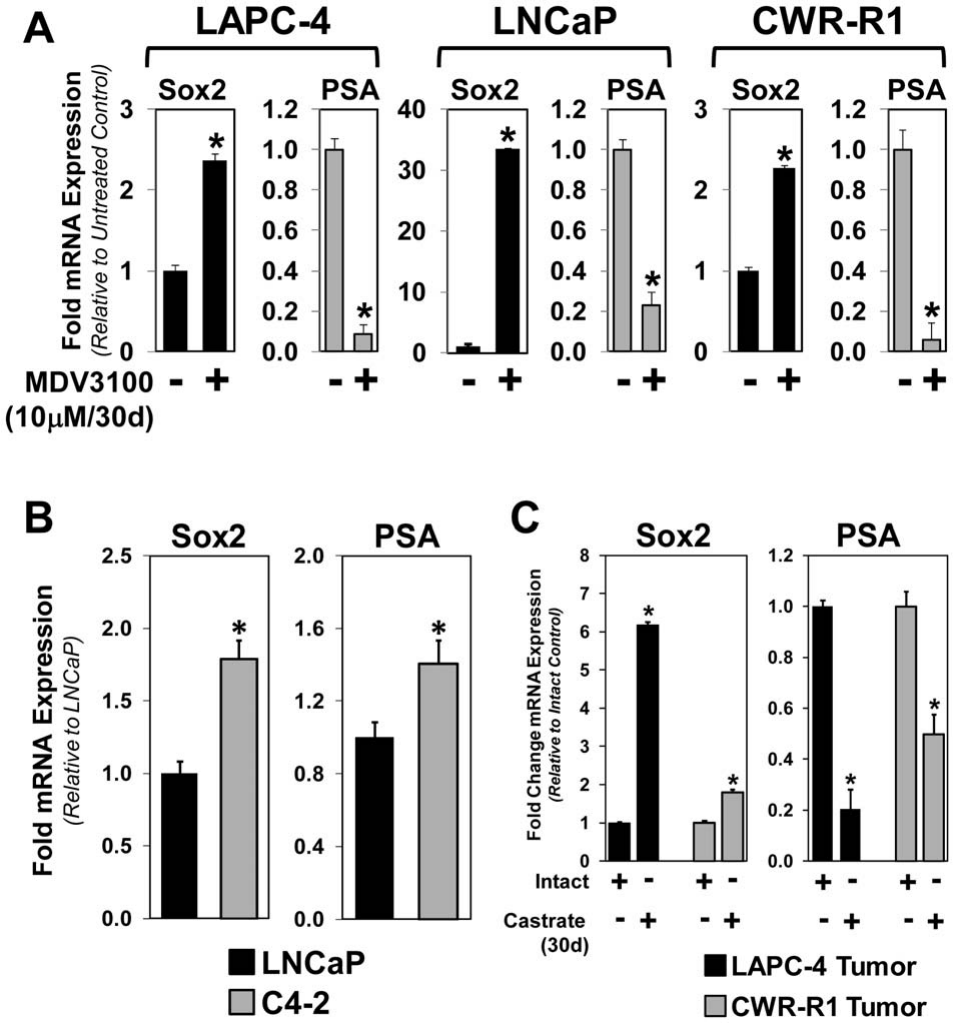
Sox2 expression is increased in MDV3100-resistant prostate cancer cells and castration-resistant prostate tumors. **A**) To test whether Sox2 expression is associated with resistance to AR pathway inhibition, we developed a series of prostate cancer cell lines that were resistant to MDV3100. After 30 days of continuous treatment, MDV3100-resistant lines expressed significantly higher Sox2 as measured using qPCR (*p<0.05). AR-mediated PSA expression was also significantly reduced in these lines. **B**) Comparison between the castration-sensitive LNCaP and isogenic castration-resistant C4-2 cell lines documents a significant increase in Sox2 expression in castration-resistant C4-2 cells (*p<0.05). **C**) Increased Sox2 expression in castration-resistant xenografts of LAPC-4 and CWR-R1 tumors. Tumors were allowed to establish and then host mice were castrated; 30 days later castration-resistant tumors were harvested and total mRNA analyzed for Sox2 and PSA. These data show increased Sox2 within castration-resistant LAPC-4 and CWR-R1 tumors (*p<0.05; data represents quantitation from multiple tumor specimens).

The detection and AR regulation of Sox2 in castration-resistant CWR-R1 cells suggested that Sox2 expression increases in response to host castration or AR inhibition and thus promotes castration-resistant prostate cancer cell growth. We tested this possibility using three different approaches. First, LAPC-4, LNCaP, and CWR-R1 cells were cultured under continuous AR inhibition using the AR antagonist MDV3100 for 30 days in culture. These MDV3100-resistant isogenic lines were then tested for AR activity using PSA expression and Sox2 levels. In all three cell lines, the levels of Sox2 become detectable within the MDV3100-resistant lines (**Figure 6A**). As expected, there is a significant decrease in PSA expression under continuous AR inhibition with MDV3100 (**Figure 6A**). Second, we analyzed the expression of Sox2 between the castration-sensitive LNCaP cells and the castration-resistant isogenic derivative C4-2 cells [52]. These data show that C4-2 cells express detectable levels of Sox2 compared to LNCaP cells, albeit at levels below detection via standard western blotting. Third and finally, we derived *in vivo* xenograft tumors from LAPC-4 and CWR-R1 cells in intact male mice, and once tumors were established the tumor-bearing mice were castrated to promote tumor regression and castration-resistant tumor re-growth. In this approach, xenograft tumors were allowed to form in a hormonally-intact male mouse until they reached a volume of 0.5 cm^3^, and then mice were castrated. These tumors initially regressed, but then re-grew in the castrated host until the endpoint 30 days after host castration. In the castration-sensitive LAPC-4-derived tumors, there is a significant increase in Sox2 expression compared to intact controls, with a marked decrease in PSA expression (**Figure 6C**). Likewise, in CWR-R1-derived tumors there is a significant increase in Sox2 expression 30 days after host castration (**Figure 6C**). These data demonstrate that, in response to AR signaling inhibition either via chronic anti-androgen or host castration, there is a significant increase in Sox2 expression within isogenic castration-resistant prostate cancer cells. These data also imply that Sox2 may function to confer castration-resistant tumor cell survival and growth.

### Sox2 is Sufficient to Promote Castration-Resistant Tumor Growth

**Figure 7:**
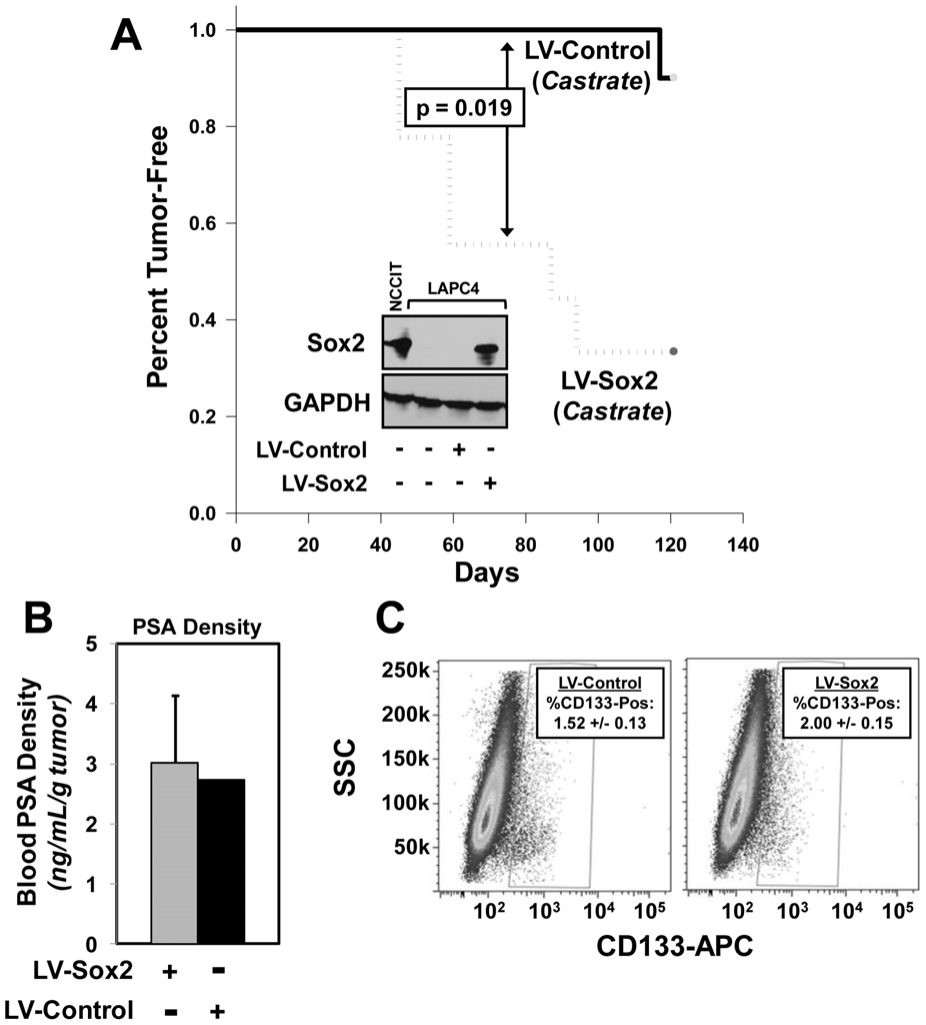
Sox2 expression promotes castration resistant prostate cancer tumor formation. **A**) Ectopic expression of Sox2 in castrate-sensitive LAPC-4 (LV-Sox2, n = 10 mice) cells is sufficient to promote tumor take in castrated male nude hosts compared to control mice (LV-Control, n = 10) (p = 0.019). Inset: western blot documenting ectopic lentiviral Sox2 expression in LAPC-4 cells. GAPDH was used as a loading control. NCCIT cells are used as a Sox2-positive control. **B**) Castrated LAPC-4-Sox2 versus LAPC-4-Control tumors do not have differences in serum PSA density. These data support that Sox2 expression does not confer a less-differentiated tumor phenotype. PSA density is the ng/mL total PSA per gram tumor. **C**) Expression of Sox2 in LAPC-4 does not significantly increase the percentage of rare CD133-positive cells, which are thought to be putative cancer stem/initiating cells.

The expression of Sox2 in the castration-resistant CWR prostate cancer cell lines and the increased expression of Sox2 in isogenic castration-resistant cells inferred that Sox2 may confer castration resistance to prostate cancer cells. To test this, we ectopically expressed Sox2 in the castration-sensitive LAPC-4 prostate cancer cell line. LAPC-4 cells are representative of a majority of advanced prostate cancers as they express elevated levels of wild-type AR [53], secrete detectable PSA, and require Androgen-Receptor signaling for their survival and growth [39], [40], [54], [55]. Thus, LAPC-4 cells do not efficiently form tumors in castrated male nude murine hosts [39], allowing us to test whether Sox2 expression was sufficient to promote castration-resistant tumor formation. Indeed, ectopic expression of lentiviral Sox2 was sufficient to significantly increase LAPC-4 tumor formation in castrated male nude hosts (**Figure 7A**). This increase in tumor take was not due to changes in cell proliferation *in vitro* since there were no measurable differences in cell cycle distribution between Sox2-expressing and control LAPC-4 cells in culture (data not shown). To test whether Sox2 promoted a less-differentiated tumor phenotype, we measured the quantity of circulating PSA in LAPC-4-Sox2 tumor bearing animals (**Figure 7B**). It should be noted that rodents do not express PSA; therefore any detectable circulating PSA expression can be attributed to the growing human xenograft. Circulating PSA was still detectable in LAPC-4-Sox2 tumor-bearing mice (2.94±0.42 ng/mL), even with castrate levels of host testosterone. Moreover, the circulating PSA of LAPC-4-Sox2 tumor bearing mice was not different than the single LV-Control tumor-bearing host (**Figure 7B**). These data document that Sox2 is sufficient to promote castration-resistant tumor formation in AR-dependent prostate cancer cells, but does not appear to promote a less differentiated and PSA-negative tumor phenotype.

One possibility for the increased castration-resistant tumor formation of the LAPC-4-Sox2 cells is that Sox2 expression may promote a cancer stem/initiating tumor phenotype that would promote tumor xenograft formation. A common feature of many tumor initiating/stem cells is the cell surface expression of CD133; previous studies have shown that CD133-positive prostate cancer cells more efficiently establish as tumor xenografts and are present as rare populations in prostate cell lines [31], [56]. Comparison of Sox2-positive vs. control LAPC-4 cells, however, demonstrates that the percentage of CD133-positive cells does not change when Sox2 is expressed (**Figure 7C**). Another functional *in vitro* assay for a stem cell phenotype is a floating spheroid culture assay. This assay yielded no significant change in spheroid formation between LAPC-4 (LAPC-4-GFP: mean = 24.7, stdev = 7.6; LAPC-4-Sox2: mean = 23.3, stdev = 10.0 [p = 0.863]) and LNCaP (LNCaP-GFP: mean = 776.7, stdev = 321.0; LNCaP-Sox2: mean = 582.0, stdev = 103.1 [p = 0.374]) cells transduced with Sox2 or Control GFP. Thus, ectopic expression of Sox2 does not appear to confer properties characteristic of cancer stem cells.

### Loss of Sox2 Expression in Castration-Resistant Prostate Cancer Cells Inhibits Cell Growth

**Figure 8:**
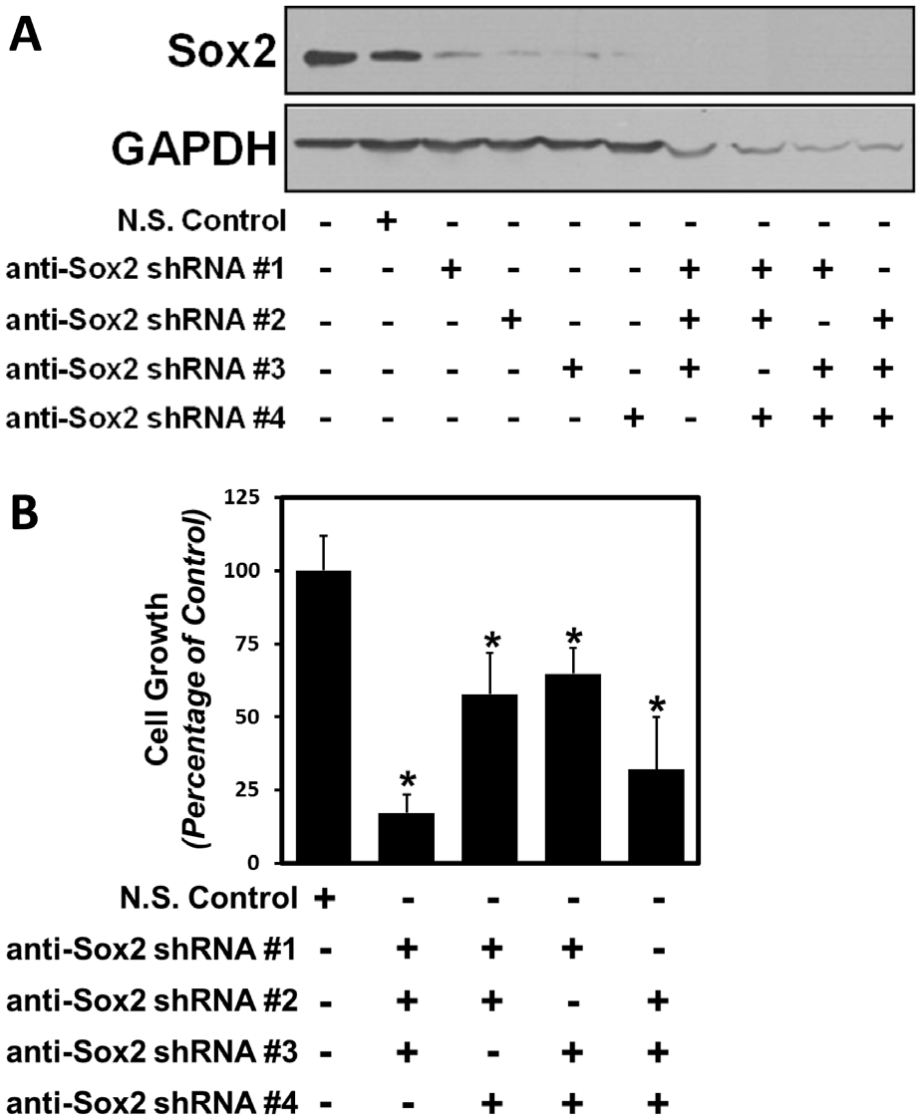
Sox2 expression is necessary for the growth of castration-resistant prostate cancer cells. **A**) Depletion of Sox2 protein expression using four different lentiviral shRNA constructs. To test the impact of inhibiting Sox2 expression in castration-resistant CWR-R1 cells, we used a series of shRNAs that targeted different regions of the Sox2 mRNA transcript. These data show that each shRNA sequence results in decreased Sox2 protein expression after 72 hours, and combinations of three were used to obtain levels below detection while controlling for potential off-target (i.e. non-Sox2 specific) effects. A non-silencing shRNA (NSC) control was used in comparison. GAPDH was used as a loading control. **B**) Decreased expression of Sox2 results in significant growth inhibition. Cell growth was measured using MTT reduction after five days in complete media. In all four instances of Sox2 knockdown, cell growth was significantly diminished compared to the non-silencing control.

To test whether Sox2 expression was necessary for prostate cancer cell survival or castration-resistant growth, we targeted Sox2 expression using shRNA expression against Sox2. Using a set of four distinct Sox2 targeting sequences, along with a non-silencing control, we demonstrate measureable decreases in Sox2 protein expression in CWR-R1 cells after 72 hours using all four shRNA targeting sequences (**Figure 8A**). Combinations of the different shRNAs were used to maintain knock-down of Sox2 expression in order to address its effect on cell growth; as transduction with single shRNA sequences failed to produce cells with continually decreased Sox2 expression compared to a non-silencing control (data not shown). The combinations of three of the four different shRNA targeting sequences were used for greater knock-down efficiency, as well as to minimize the off-target effects of each of the individual shRNA constructs [57]. As a result of decreased Sox2 protein expression, there is a significant inhibition of cell growth over seven days in the presence of complete media (**Figure 8B**). All of the different combinations of shRNA targeting sequences produced cells with decreased viability and greater Sox2 knock-down. The slight decrease in GAPDH protein levels (**Figure 8A**) suggests that these cells are already demonstrating decreased viability in response to Sox2 knock-down even by 72 hours after lentiviral infection. These data demonstrate that Sox2 is essential for the survival of castration-resistant CWR-R1 prostate cancer cells.

### Sox2 Regulates Different Genes in Prostate Cells than in Embryonic Stem Cells

**Figure 9:**
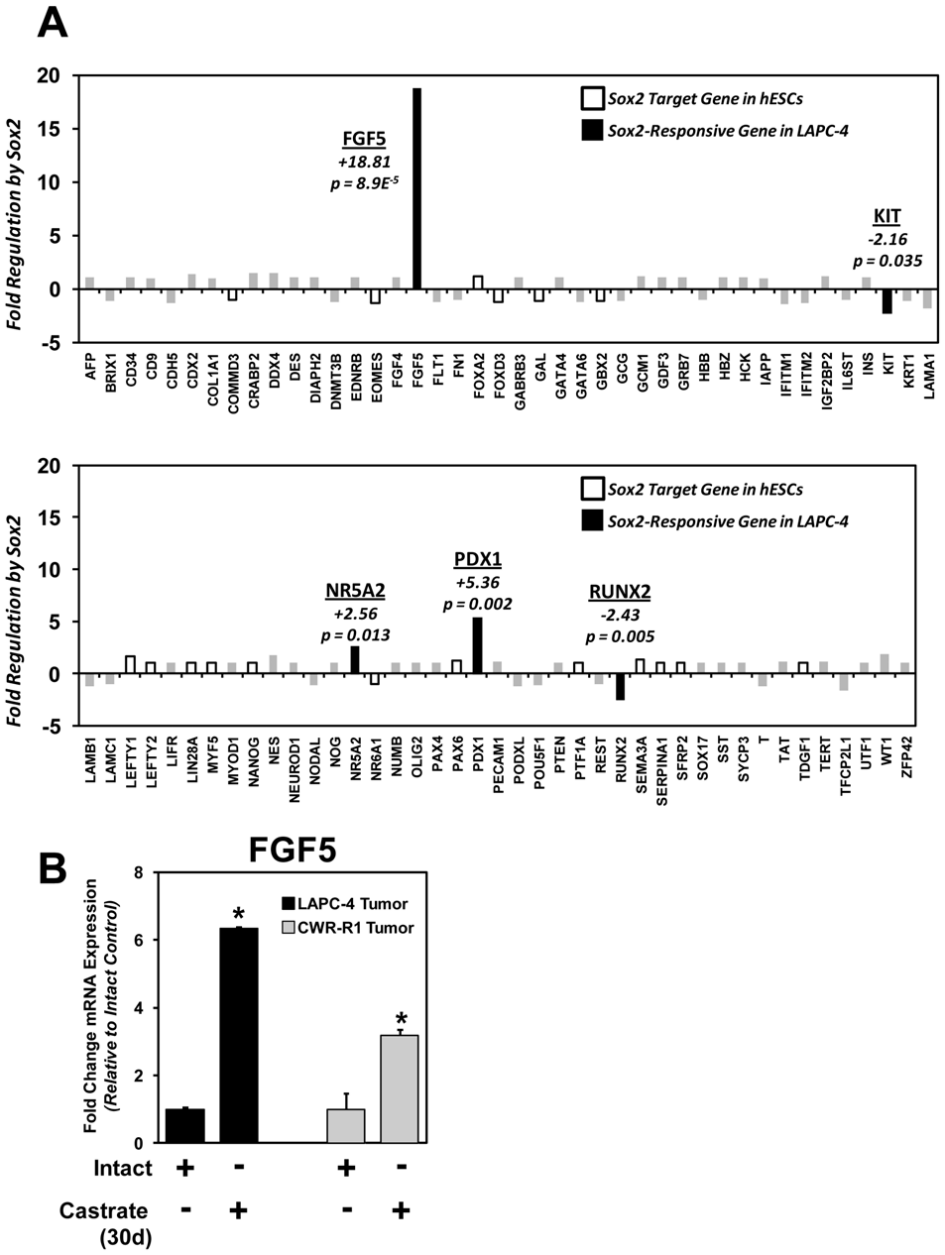
Sox2 expression is associated with the expression of FGF5 and not established human embryonic stem cell Sox2 target genes. **A**) The expression of 83 embryonic stem cell-associated genes was analyzed via quantitative real-time PCR analyses to identify Sox2-associated gene changes in LAPC-4-Sox2 cells (compared to LAPC-4-Control cells). Open bars represent genes that are previously identified Sox2-target genes in hESCs (Boyer et al. 2005). No changes in any of these 18 genes were associated with Sox2 expression in LAPC-4 cells. Rather, significant changes in expression were detected for FGF5, Kit, NR5A2, PDX1, and RUNX2 (black bars). **B**) Elevated expression of FGF5 in castration-resistant LAPC-4 and CWR-R1 tumors (*p<0.05; same mRNA as described in Figure 6C).

The ability of Sox2 to promote castration-resistant tumor growth of AR-dependent LAPC-4 prostate cancer cells without impacting CD133 or PSA expression suggested that Sox2 does not promote the re-expression of embryonic stem cell genes thereby promoting a “stemness” phenotype similar to that observed in tumor initiating/stem cells. However, since LAPC-4 do endogenously express the Sox2 co-regulator Nanog (**Figure 4A**), it is possible that ectopically-expressed lentiviral Sox2 may be partnering with Nanog to promote the expression of common embryonic stem cell target genes or modulating Nanog expression [21]. Previous studies have identified Sox2 target genes in human embryonic stem cells using Chromatin-IP (ChIP) and targeted knockdown [21], [58]. In hESCs, Sox2 binds the promoters of 1289 target genes, many of which are co-regulated with Oct4 and/or Nanog [21]. The lack of Oct4 and Nanog expression in Sox2-positive CWR cells and lack of Oct4 expression in LAPC-4 cells, however, indicates that Sox2 may regulate a different set of genes in normal and malignant prostate epithelial cells. To test whether Sox2 promoted the expression of genes associated with hESCs, we conducted quantitative real-time PCR (qPCR) analyses of 83 established embryonic stem cell genes, 18 of which were documented to be targeted by Sox2 in hES cells [21]. Comparison of Sox2-positive versus control LAPC-4 cells reveals that none of the 18 of the stem cell-associated genes known to be regulated by Sox2 in hES cells, including Nanog, are differentially expressed (**Figure 9A**). Instead, Sox2 is associated with the expression of previously undefined Sox2-targets FGF5, NR5A2 and Pdx1, while it inhibits the expression of c-Kit and Runx2. By far the most significant change is FGF5 (Fibroblast Growth Factor 5; RefSeq NM_004464). Like other FGFs, it is involved in a broad array of mitogenic and morphogenic processes, but its expression appears to be restricted to embryonic development. To date, little is known about the role of FGF5 in the prostate or its involvement in prostate cancer or castration-resistant tumor growth. To test whether FGF5 expression increases in castration-resistant tumor xenografts, we analyzed the castration-resistant LAPC-4 and CWR-R1 tumors described previously (**Figure 6C**); these data demonstrate that in addition to an increase in Sox2 expression there is a significant increase in FGF5 expression within castration-resistant tumor xenografts (**Figure 9B**). This finding demonstrates that in castration-resistant LACP-4-Sox2 cells, expression of Sox2 is not associated with the expression of defined Sox2 targets genes in human embryonic stem cells, including Nanog. Rather, increased expression of Sox2, either by ectopic lentiviral expression or selection of castration-resistant tumors, results in increased FGF5 expression.

## Discussion

Here we show that Sox2 is expressed in both normal and malignant prostate epithelial cells and in both cases the expression of Sox2 decreases upon AR signaling activation. The function of Sox2, however, appears to not be via the regulation of known embryonic stem cell Sox2 target genes, but rather through a novel set of Sox2 gene targets. Data reported here is significant for four major reasons. First, our studies identify a subset of tumors that are uniformly Sox2-positive; such tumors may have enhanced capabilities for disease progression and acquisition of castration resistance. Second, the observation that only a portion of prostate basal epithelial cells are Sox2-positive suggest that prostate tumors may arise from either a Sox2-positive or Sox2-negative basal epithelial cell, and that these tumors of different cell origin may display unique clinical progression characteristics [35]. Third, the AR-mediated repression of Sox2 provides a potential new mechanism for castration-resistance, whereby release of AR-mediated Sox2 repression during androgen-deprivation therapy restores Sox2 expression, resulting in increased FGF5 expression and an as yet undefined survival advantage. Fourth and finally, the function of Sox2 to promote castration-resistant tumor growth appears to not be via re-activation of established hES pathways, but rather through a novel mechanism independent of Nanog and Oct4.

The uniform expression of Sox2 in a subset of hormone naïve prostate tumors and the majority of castration-resistant metastases analyzed suggest multiple potential pathologic roles for Sox2 in prostate tumor initiation and progression. First, Sox2-positive tumors may confer a worse overall disease prognosis thus implicating positive Sox2 tumor expression as a potential biomarker for discerning poor prognosis tumors. Second, our data demonstrating AR-mediated transcriptional repression of Sox2 in normal and malignant prostate cells imply that there may be deficiencies in AR signaling within Sox2-positive hormone naïve tumors. Third and finally, elevated expression of Sox2 in the majority of castration-resistant metastases and tumor xenografts implicates a role for Sox2 and its target genes such as FGF5 in promoting castration resistance. Thus, inhibiting the function of Sox2 and/or its target genes may have the potential to aid in the treatment of prostate cancer, and prevent progression to a castration-resistant state. Further studies, however, are required to test these hypotheses.

Our understanding of the function of Sox2 in other malignancies is rapidly expanding, and Sox2 expression has been documented among lung, breast, hepatocellular, lung, and esophageal cancers [20], [59], [60]. In hepatocellular carcinomas, the expression of Sox2 confers greater disease aggressiveness, as patients bearing Sox2-positive tumors demonstrated significantly shorter survival intervals [61]. In lung and esophageal squamous cell carcinomas, Sox2 does in fact promote an embryonic stem cell gene expression signature [59], but the overall clinical impact of Sox2 expression is unresolved [59], [62]. Data presented here indicate a unique function for Sox2 in human prostate tissues and tumors. This is due to the observation that the Sox2 transcriptional co-factors Oct4 and Nanog are not co-expressed in Sox2-positive normal PrEC cultures and castration-resistant CWR cells, and that expression of Sox2 in Nanog-positive LAPC-4 cells does not result in increased expression of hESC Sox2-gene targets. These data imply that, in adult prostate cells and tumors, Sox2 may cooperate with as yet undefined transcriptional co-factors to regulate castration-resistance and tumor progression.

The expression of Sox2 in a non-stem cell context without Nanog or Oct4 also raises critical questions about its epigenetic regulation within cancer. Variations in the levels of pluripotency factors have been implicated in governing cell fate by exhibiting differential binding, occupancy and regulation of co-activators/co-repressor proteins in specific promoters; these variations result in lineage specific differentiation of cells [63], [64]. Thus, the lack of detectable transcriptional partners of Sox2 could impact occupation of available promoters and produce the target genes that our data suggests are quite different than those regulated during pluripotency in embryonic stem cells [65], [66]. The identification of these different Sox2 target genes and Sox2 binding partners have the potential to highlight novel and clinically targetable pathways to prevent and/or treat castration-resistant prostate tumor formation and metastatic growth. In fact, recent studies have investigated the use of artificially engineered exogenous proteins that aim to silence the Sox2 promoter; these data are exciting given that non-nuclear receptor transcription factors are notoriously hard to target therapeutically [67].
